# Client predictors of therapy dropout in a primary care setting: a prospective cohort study

**DOI:** 10.1186/s12888-023-04878-7

**Published:** 2023-05-24

**Authors:** Elin Hanevik, Frida M. G. Røvik, Tormod Bøe, Marit Knapstad, Otto R. F. Smith

**Affiliations:** 1 Søndre Oslo DPS, Helga Vaneks Vei 6, 1281 Oslo, Norway; 2 Rask Psykisk Helsehjelp, Bydel Ullern, Hoffsveien 48, 0377 Oslo, Norway; 3 Department of Psychosocial Science, The University of Bergen, Christies Gate 12, 5015 Bergen, Norway; 4 RKBU Vest, NORCE Norwegian Research Centre, Bergen, Norway; 5 Department of Health Promotion, Norwegian Institute of Public Health, Zander Kaaes Gate 7, 5015 Bergen, Norway; 6 Centre for Evaluation of Public Health Measures, Norwegian Institute of Public Health, Oslo, Norway; 7 Department of Teacher Education, NLA University College, Pb 74 Sandviken, 5812 Bergen, Norway

**Keywords:** Dropout, Cognitive Behavioral Therapy, Prompt Mental Health Care, Improving Access to Psychological Therapies

## Abstract

**Background:**

Therapy dropout poses a major challenge. Considerable research has been conducted on predictors of dropout, however none in the context of primary mental health services in Norway. The purpose of this study was to investigate which client characteristics can predict dropout from the service Prompt Mental Health Care (PMHC).

**Methods:**

We performed a secondary analysis of a Randomized Controlled Trial (RCT). Our sample consisted of 526 adult participants receiving PMHC-treatment in the municipalities of Sandnes and Kristiansand, between November 2015 to August 2017. Using logistic regression, we investigated the association between nine client characteristics and dropout.

**Results:**

The dropout rate was 25.3%. The adjusted analysis indicated that older clients had a lower odds ratio (*OR*) of dropping out compared to younger clients (*OR* = 0.43, [95% CI = 0.26, 0.71]). Moreover, clients with higher education had a lower odds ratio of dropping out compared to clients with lower levels of education (*OR* = 00.55, 95% CI [0.34, 0.88]), while clients who were unemployed were more likely to drop-out as compared the regularly employed (*OR* = 2.30, [95% CI = 1.18, 4.48]). Finally, clients experiencing poor social support had a higher odds ratio of dropping out compared to clients who reported good social support (*OR* = 1.81, [95% CI = 1.14, 2.87]). Sex, immigrant background, daily functioning, symptom severity and duration of problems did not predict dropout.

**Conclusion:**

The predictors found in this prospective study might help PMHC-therapists identify clients at risk of dropout. Strategies for preventing dropout are discussed.

## Background

### The mental health status in Norway

In Norway, the yearly prevalence of mental disorders in the population is around 20% [33]. This indicates that 1 in 5 adults will have a mental disorder in any given year. Anxiety, depression, and drug addiction are the most common disorders.

Anxiety and depression are often reported as reasons for reduced ability to work, sick leave and disability benefits in Norway [32]. Among those who received disability benefits in 2016, 36.8% were allocated this due to a primary diagnosis of a mental or behavioral disorder. Overall, this represented the largest proportion of people receiving disability benefits [32].

The Norwegian health care system is divided into different units, called primary, secondary and tertiary services. The primary services often have a preventive and health promoting mandate. This includes all services clients can use without a referral, and are often free of charge. Treatment in secondary services requires a referral from the primary service, and clients are admitted based on a higher symptom severity. Tertiary health services require a referral from the secondary service as they are more specialized to certain disorders.

### Prompt Mental Health Care

Prompt Mental Health Care (PMHC), in Norwegian called Rask Psykisk Helsehjelp (RPH), is a primary care treatment model based on Cognitive Behavioral Therapy (CBT). PMHC is based on Improving Access to Psychological Therapies (IAPT), a program implemented by the UK Government in 2008. IAPT has shown solid treatment results and has proven to give mass public benefits [5]. Today, there are similar services to IAPT in a growing number of countries, such as Norway, Australia, Japan, and Sweden [49].

An important goal of PMHC is to improve access to evidence-based treatment for adults with mild to moderate anxiety and depression, sleep problems and emerging substance use problems [46]. A secondary goal is to enhance work participation. PMHC is easily accessible because it is free, situated in the local community, and approachable without a referral from a general practitioner (GP). The treatment is based on a *mixed care model*, entailing application of a mix of treatment modalities with various intensity. These modalities range from low-intensity guided self-help, courses and groups to more high-intensity, short term individual therapy. The services are provided by interdisciplinary teams educated in CBT [46]. Evaluations have shown solid effects of PMHC [24, 25, 40, 46].

### Therapy dropout

Despite well-documented recovery effects, it is a fact that therapy does not bring desirable results for everyone [6, 15, 50]. A considerable proportion of clients terminate therapy prematurely for a number of reasons. This group is often referred to as dropouts [1, 13, 48, 52]. Dropout has become a field of interest within research over the past fifty years, with hopes of implications that can provide meaningful and efficient therapy courses for more people.

Dropout is defined in various ways across the literature [1, 13, 48, 52]. Across definitions, dropout is often operationalized in a threefold manner, highlighting one or more of the following aspects: 1) The number of sessions attended, 2) Premature termination, understood as termination prior to recovery, or 3) Unilateral termination, understood as lack of therapist collaboration on the decision of termination.

Meta-analyses and literature reviews have found the average prevalence of dropout to vary due to differences in definitions, study designs, and service settings [10, 52, 54]. For psychotherapy in general, meta-analyses have shown a mean average dropout rate of approximately 19–46% [48, 52]. Looking at CBT studies exclusively, meta-analyses and literature reviews have reported an average dropout rate between 15–26% [13, 19],Linardon et al., 2018, [41]. Within the IAPT treatment setting, an unpublished meta-analysis found an average dropout rate of 31% across all studies [16]. There were notable differences between the dropout rates reported in the studies, ranging from approximately 10–50%.

Dropout can have extensive consequences for the client and the service. First of all, it decreases the chances of clinical recovery, in terms of higher symptom severity for dropouts at termination compared to completers [4, 14, 43, 54]. Residual subthreshold symptoms are a risk factor for relapse, which increases the chance of long-term poor outcome and several courses of therapy [4, 34, 54]. There are also negative consequences for the national health care system and the local services in terms of lost time, resources, and economic loss [10, 30]. Notably, dropout is not always equivalent to negative client outcomes. It seems that for some clients a few sessions can be enough to feel better and subsequently drop out [30].

### Previous research on predictors of dropout

A number of predictors of dropout have been identified in the literature, however, somewhat inconsistent [1, 41, 48, 52]. There is evidence that a considerable amount of the client dropout variance is explained at the therapist level with findings ranging from 5.7%-12.6% [42, 55]. Further, therapeutic alliance is found to be related to dropout [17, 22, 45], and some claim the therapeutic alliance to be more predictive than client and therapist factors separately [52]. Additionally, dropout can to some extent be predicted by differences between services. Di Bona et al. [10] and Reneses et al. [39] reported that belonging to different municipalities or being allocated to different services provided different dropout rates. Nevertheless, the largest body of research has been done on clinical and sociodemographic client predictors of dropout.

#### Clinical factors

High symptom severity has been presented as a predictor of client dropout, especially high levels of depression and anxiety [3, 13, 21, 42, 51]. Studies have interestingly also found low symptom severity and high daily function to be a predictor of dropout [10, 12, 54]. The findings on low and high symptom severity as predictors of dropout might represent a bi-modality. The two opposites can potentially lead to clients perceiving treatment as either unmanageable or unnecessary because one is too well.

Similarly to the bi-modality of symptom severity, duration has been found to be predictive either if the episode had persisted for a long time (> 2 years) or quite a short time (< 1 month) [10].

#### Sociodemographic factors

Most meta-analyses and literature reviews conclude with inconsistent and mixed results for sex as a predictor [1, 48, 54]. A dominant body of research has found that younger age is predictive of dropout [11, 12, 22, 39, 42, 54].

In studies by Barrett et al. [1] and Fenger et al. [12], lower socioeconomic status (SES) was presented as the most important demographic predictor of dropout. This is found across several SES indicators, including economic deprivation or poverty [3, 14, 52], lower levels of education [12, 23, 41, 52], high levels of social deprivation [18, 44].

Unemployment has also been identified as a predictor of dropout [12, 14]. Saxon et al. [42] found that unemployment was the strongest predictor investigated of both dropout and deterioration. Interestingly, Zieve et al. [54] did not find unemployment to be a predictor of dropout in a private clinical setting. Fenger et al. [12] found that clients on sick leave had an increased frequency of treatment show-up. It is speculated that sick leave can decrease the chance of dropout because the client’s daytime schedule is more open for treatment sessions [12]. This is supported by the fact that work commitment is commonly mentioned as a reason for dropout [3, 17].

Studies have provided mixed results for immigrant background as a predictor of dropout. Some studies have found an association between immigrant background and dropout [1, 8, 51, 52], however the findings are not consistent across the field.

### The aim of the study

As demonstrated, there exists a large body of research on dropout from psychotherapy. The findings on predictors of dropout are somewhat inconsistent, especially related to client factors. There is a growing field of research that documents and supports the effect of health prevention through primary care services [38]. However, there is limited research on dropout from primary mental health services. With data provided from The Norwegian Institute of Public Health (NIPH), our study aimed to investigate whether a number of client factors could predict dropout from the service PMHC in Norway. No research on dropout had previously been conducted in this service setting. We focused exclusively on client factors, as our dataset consisted of client baseline characteristics. Based on the literature, we selected the following nine factors from the dataset we had at hand: age, sex, level of education, work status, immigrant background, social support, symptom severity, duration of problems, and daily function.

## Methods

Data was provided by NIPH. It was obtained from the PMHC treatment arm of a pragmatic Randomized Controlled Trial (RCT) conducted in two Norwegian municipalities, Sandnes and Kristiansand. We looked into predictors of dropout among those who received the intervention, thereby making this a prospective cohort study design. The descriptions of subjects, materials, and methods were first described in the primary evaluation of the RCT by Knapstad et al. [24].

### Data collection and procedure

Participants in this study were recruited between November 2015 and August 2017 [24]. The trial sites were found to be relatively similar to each other as well as representative for the Norwegian population on several sociodemographic variables, for instance, rates of immigrant background, higher education, and unemployment [24].

Psychologists had professional responsibility for the service at each site. Ten therapists were included in the current study. The number of clients per therapist ranged from eight to 90 clients (m = 52). The majority of clients started with a four-session psychoeducational course. Low-intensity self-help programs were to a limited extent accessible throughout the trial period. Most clients received only low-intensity treatment in terms of group-based psychoeducation (36.5%) or a combination of low and high-intensity interventions (33%). Furthermore, 29.4% primarily received high-intensity treatment. Only 1% received guided self-help [28]*.*

### Recruitment and participants

Information about the study was conveyed both through an information letter from NIPH to all GPs in the area and directly from the services at local GP association meetings. Citizens could get information about the study from their GP, through the municipality web page, local newspapers, and local radio. People who contacted PMHC in Sandnes or Kristiansand got an appointment for an initial assessment. This assessment consisted of a clinical interview to evaluate the client's mental health problems and motivation for treatment, in addition to providing information about the study.

There were predefined inclusion and exclusion criteria to evaluate participants’ eligibility for PMHC during the trial period. The criteria were supposed to resemble ordinary care. The primary inclusion criterion was anxiety and/or mild to moderate depression. The Patient Health Questionnaire (PHQ-9) and Generalized Anxiety Disorder scale (GAD-7) were used as screening instruments with predetermined cut-offs (PHQ-9 >  = 10 and/or GAD-7 >  = 8) [24]. Upper cut-offs for excision of severity were not predefined as severity was also based on clinical judgment in the clinical interview. Further requirements were a minimum age of 18 years, place of residence in the relevant municipalities, and basic Norwegian language proficiency.

People were excluded if they met the criteria of more profound mental problems such as eating disorder, severe suicidal risk, bipolar disorder, severe depression, incapacitating anxiety, psychotic symptoms, substance abuse, or personality disorder. Another exclusion criteria was two or more previous attempts at treatment in the secondary services, without satisfactory effect. People with serious physical health problems as their primary challenge were also excluded. Those not considered eligible for PMHC were referred to their GP, secondary services, or other services suitable for their main challenge.

Those who met the inclusion criteria were asked to participate, gave their written consent and registered on a secure online data portal. The portal was developed by the Norwegian Social Science Data Services (NSD) and was used to collect all data and questionnaires from clients and therapists. It was also used to randomize the clients to either PMHC treatment or treatment as usual (TAU) [24, 40]. There were 774 participants who were included in the trial, whereof 526 were randomized to PMHC treatment [24, 40]. Participant data from the PMHC group was used for the analysis in this paper.

### Measures

#### Outcome measure

*Dropout* in the context of this study was defined as dropout occurring before completing six treatment sessions. Six sessions were chosen as this is regarded as the minimum number of recommended sessions for the treatment of anxiety and depression in IAPT [31]. Clients who achieved their treatment goals prior to six sessions and terminated in agreement with the therapist, were not classified as dropouts. Therapists reported completion or dropout, the numbers of sessions attended, and the reasons for termination.

#### Baseline predictors

When the clients had registered, they self-reported their answers to a variety of questions in a baseline questionnaire. The questions ranged from mental and physical health to demography and lifestyle. All continuous variables were dichotomized to facilitate interpretation and to increase the clinical utility of the study results.

### Clinical variables

PHQ-9 asks the responder to evaluate nine items describing each criterion for depression based on DSM-V. The response options vary from 0 (*not at all*) to 3 (*nearly every day*), which allows a maximum sum score of 27. Caseness was defined as a minimum score of 10. A score above 14 was defined as moderate to severe symptoms of depression. The scores were coded into three different categories, namely below cut-off (0–9), mild depression (10–14), and moderate to severe depression (15–27). The variable *below cut-off* was used as a reference category. The PHQ-9 has been tested as a reliable and valid measure for making criteria-based diagnoses for depression, assessing symptom severity, and monitoring change over time [27]. The internal reliability of PHQ-9 has been measured and evaluated, showing excellent test–retest reliability and Cronbach’s α between 0.86–0.89 [27]. Cronbach’s α based on our data was 0.80.

GAD-7 measures the frequency of seven common symptoms of general anxiety. Similar to PHQ-9, the response options vary from 0 (*not at all*) to 3 (*nearly every day*). The maximum sum score is 21. Caseness was set at 8, and a score above 14 was defined as severe symptoms of anxiety. GAD scores were coded into three categories, namely below cut-off (0–7), mild-moderate anxiety (8–14), and severe anxiety (15–21). *Below cut-off* was used as a reference category. GAD-7 has been found to have good validity and reliability for measuring general anxiety. The instrument can be used both to assess symptom severity and monitor change over time [24, 47]. It has shown excellent test–retest reliability and Cronbach’s α of 0.92 [47]. Cronbach’s α based on our data was 0.83.

The Work and Social Adjustment Scale (WSAS) measures impairment of daily function by evaluating five items on a scale ranging from 0 (*not at all*) to 8 (*very severely*). The answers are based on function at work and in social relations during the last month [53]. The sum scores reported were converted to a binary variable. Scores within the highest tertile were coded as 1 (*low functional status*), while scores in the lowest two tertiles were coded as 0 (*high functional status*). WSAS has been used in former PMHC evaluations [46]. Furthermore, WSAS has comparable reliability, sensitivity, and discriminant validity to PHQ-9 and GAD-7 [53].

Duration of problems was measured in months. The variable was recoded into three categories: *less than or equal to 6 months*, *between 7 and 24 months*, and *longer than 24 months*. The middle category was used as reference based on findings from the literature review.

#### Sociodemographic variables

The sociodemographic questions were used as binary variables. These questions included sex (*female: yes/no*), higher education (*university/college: yes/no*), and immigration background (*1st or 2nd generation immigrant: yes/no*). Employment was assessed by two multiple response questions regarding current work status and source of income. Based on their answers, participants were coded into five different categories. These were *employed, employed while receiving benefits, unemployed, students and other (e.g. retirees, full disability pensionists).* The *employed* category was used as a reference category. Age was also used as a binary variable (above 30 years: yes/no) as the literature suggests that particularly younger people are at risk to drop-out. Even though there is always a degree of arbitrariness in choosing a cut-off, our observed data suggested a marked drop in the probability of dropping out after age 30.

Questions about lifestyle and social variables were also reported using binary responses. Most relevant for this analysis was the question of social support. The 3-item Oslo Social Support Scale (OSSS-3) covers the number of close confidants, the sense of concern shown by others, and perceived availability of practical help from neighbors [26]. A sum score ranging from 3 to 14 was calculated. Clients scoring 3 to 8 were coded as 1 (*low social support*), whereas those scoring 9 to 15 were coded as 0 (*medium to high social support*). Validity and reliability for OSSS-3 have been reported as satisfying [26]. Cronbach’s α of the OSSS-3 was relatively low based on our data (0.58).

### Statistical analyses

Preliminary analyses were undertaken to prepare the specific statistical techniques to address the research question. All variables were checked for errors, outliers, normality of distribution, variance, and missing data. Within the variables higher education, duration of problems, and immigrant background, we found some missing data (< 3%). Missing data were handled by listwise deletion in the regression analyses. Logistic regression was considered the most appropriate analysis as the dependent variable was dichotomous [35].

To examine possible relationships between dropout as a dependent variable and client factors as independent variables, we first did bivariate logistic regression analyses for nine variables of relevance according to the literature. Of sociodemographic variables, these were age, sex, immigrant background, work status, level of education, and social support. Of clinical variables, these were symptom severity, duration of problems, and daily function.

The independent variables reaching* p* values < 0.05 in the logistic regression analyses were subsequently included in a multivariate logistic regression model. If the strength of an association changed when included in the multivariate analysis, further analyses were conducted to understand what accounted for the variation in the outcome variable. This was done by exploring different combinations of variables using logistic regression analysis, and observing possible changes. Therapists and municipalities were included in all analyses as fixed effects. All statistical analyses were performed using IBM SPSS Statistics, version 28.0.1.0.

## Results

### Dropout

In this current study, 133 (25.3%) participants dropped out of therapy. Meanwhile, 393 (74.7%) participants completed therapy. Therapists reported the following reasons for termination of therapy for the dropout group: not being able to contact the client (36.1%), lack of motivation (19.5%), changed to other service (15.1%), unsatisfactory effect (4.5%), moving out of municipality (3%), other reasons (4.5%) and unknown (17.3%). The mean number of sessions attended for the dropout group was 2.36 (*SD* = 1.67). For the completers group it was 7.37 (*SD* = 4.5) sessions. Dropout happened most frequently between assessment and the first session (20.0%) and between the fourth and fifth sessions (21.8%).

### Baseline characteristics

Descriptive analyses of the sample can be found in Table 1. The total number of participants was 526, of whom approximately two-thirds were female. The mean age of the sample was 34.95 (*SD* = 12) and 60% of the sample were above 30 years of age. Within the sample, 12.0% had a first or second-generation immigrant background and 44.3% reported having higher education. The majority of the sample were either employed (29.5%) or employed while receiving benefits (35.7%). The rest of the sample was either unemployed (14.3%) or students (14.3%). Within the sample, 32.5% reported having poor social support.

**Table 1:** Sociodemographic and clinical characteristics of participants at baseline

Baseline characteristic	Full sample *N* = 526	Dropout *n* = 133	Completer *n* = 393
	Frequency (%)	Frequency (%)	Frequency (%)
**Sociodemographic**			
Sex: Female	343 (65.2%)	77 (57.9%)	266 (67.7%)
Aged 30 or higher	320 (60.8%)	56 (42.1%)	264 (67.2%)
Poor social support	171 (32.5%)	55 (41.4%)	116 (29.5%)
Immigrant background	63 (12%)	19 (14.3%)	44 (11.2%)
Higher educated	231 (44.3%)	41 (31.1%)	190 (48.8%)
Work status			
Employed	155 (29.5%)	31 (23.3%)	124 (31.6%)
Employed while receiving benefits	188 (35.7%)	36 (27.1%)	152 (38.7%)
Unemployed	75 (14.3%)	31 (23.3%)	44 (11.2%)
Student	75 (14.3%)	29 (21.8%)	46 (11.7%)
Other	33 (6.3%)	6 (4.5%)	27 (6.9%)
**Clinical**			
Symptoms of depression			
Below cut-off	109 (20.7%)	23 (17.3%)	86 (21.9%)
Mild	175 (33.3%)	42 (31.6%)	133 (33.8%)
Moderate-severe	242 (46.0%)	68 (51.1%)	174 (44.3%)
Symptoms of anxiety			
Below cut-off	123 (23.4%)	28 (21.1%)	95 (24.2%)
Mild-moderate	266 (50.6%)	70 (52.6%)	196 (49.9%)
Severe	137 (26%)	35 (26.3%)	102 (26%)
Symptom duration			
≤ 6 months	100 (19.0%)	21 (15.9%)	79 (20.1%)
7–24 months	140 (26.7%)	38 (28.8%)	102 (26.0%)
> 24 months	285 (54.2%)	73 (55.3%)	212 (53.9%)
Low daily function	190 (36.1%)	52 (39.1%)	138 (35.1%)

Mean age for this sample was 34.95 (*SD* = 12). Mean score for symptoms of depression (PHQ-9) was 13.9 (*SD* = 5). Mean score for symptoms of anxiety (GAD-7) was 11.3 (*SD* = 4.6)

Looking at clinical characteristics, the PHQ-9 mean was 13.9 (*SD* = 5), while the GAD-7 mean was 11.3 (*SD* = 4.6). For PHQ-9, the majority (46%) of clients scored within moderate to severe symptoms of depression. For GAD-7, the majority (50.6%) of clients scored within mild to moderate symptoms of anxiety. Most of the sample had experienced their mental health problem for longer than six months (85.9%). A group of 36.1% reported experience of low daily function.

### Baseline characteristics predicting dropout

Results from the first bivariate logistic regression analyses are presented in Table 2. There were significant independent associations between dropout and younger age, poor social support, lower levels of education, and being a student (all *p*-values < 0.05).

**Table 2:** A Bivariate logistic regression analysis predicting dropout from sociodemographic and clinical variables

Predictor	*B*	*SE*	Wald	*df*	*p*	*OR*	95% CI for *OR*	
							Lower	Upper
**Sociodemographic**								
Aged 30 or higher	-1.02	.22	22.10	1	< .001	.36	.23	.55
Sex: Female	-.31	.22	1.98	1	.16	.74	.48	1.13
Higher educated	-.89	.23	15.30	1	< .001	.41	.26	.64
Immigrant background	.21	.31	.45	1	.50	1.23	.67	2.26
Poor social support	.60	.22	7.49	1	.006	1.83	1.19	2.81
Work status^a^								
Employed while receiving benefits	.01	.28	.00	1	.96	1.01	.58	1.77
Unemployed	1.01	.33	9.44	1	.002	2.75	1.44	5.24
Student	.77	.33	5.50	1	.02	2.16	1.14	4.10
Other	-.17	.51	.11	1	.74	.84	.31	2.31
**Clinical**								
Symptoms of depression^a^								
Mild-moderate	.09	.31	.09	1	.77	1.10	.60	2.00
Severe	.26	.29	.80	1	.37	1.30	.74	2.28
Symptoms of anxiety^a^								
Mild-moderate	.08	.27	.92	1	.76	1.09	.64	1.84
Severe	-.08	.31	.07	1	.80	.92	.51	1.69
Symptom duration								
≤ 6 months	-.24	.32	.53	1	.47	.79	.42	1.49
> 24 months	-.20	.25	.66	1	.42	.82	.50	1.33
Low daily function	.13	.22	.34	1	.56	1.14	.74	1.74

*OR* Odds ratio, *CI* Confidence interval. Municipalities and therapists were included as fixed effects. Number of participants = 526. Significance level set to *p* < .05

^a^Reference categories—work status: employed; symptoms of depression and anxiety: non-clinical symptom levels (below cut-off); symptom duration: 7 – 24 months

Table 2 shows that participants over 30 had a lower odds ratio of dropping out relative to participants under 30 (*OR* = 0.36, [95% CI = 0.23, 0.55]). Participants with higher education had a lower odds ratio of dropping out compared to those with lower levels of education (*OR* = 0.41, [95% CI = 0.26, 0.64]). Concerning work status, participants reporting to be unemployed or a student had a higher odds ratios of dropping out compared to those who were in regular work (*OR* = 2.75, [95% CI = 1.44, 5.24] resp. *OR* = 2.16, [95% CI = 1.14, 4.10]). Participants reporting poor social support were more likely to drop out compared to those who reported good social support (*OR* = 1.83, [95% CI = 1.19, 2.81]). The variables identified as significantly associated with dropout in the logistic regression analyses were subsequently included in the multivariate model.

Results from the multivariate analysis are presented in Table 3. Younger age, being unemployed, poor social support, and lower levels of education remained significant predictors of dropout (all *p*-values < 0.05), while being a student did not.

**Table 3:** Multivariate logistic regression analysis predicting dropout from therapy

Predictor	*B*	*SE*	Wald	*df*	*p*	*OR*	95% CI for *OR*	
							Lower	Upper
Age 30 or higher	-.84	.25	11.09	1	< .001	.43	.26	.71
Work status^a^								
Employed while receiving benefits	.08	.30	.07	1	.80	1.08	.60	1.94
Unemployed	.83	.34	5.98	1	.02	2.30	1.18	4.48
Student	.35	.36	.98	1	.32	1.42	.71	2.86
Other	-.19	.55	.12	1	.73	.83	.29	2.42
Poor social support	.59	.24	6.37	1	.01	1.81	1.14	2.87
Higher education	-.60	.24	6.09	1	.01	.55	.34	.88

*OR* Odds ratio. *CI* Confidence interval. Municipalities and therapists were included as fixed effects. Number of participants = 520. 6 missing cases. The multivariate model was statistically significant χ^2^ (15, *N* = 520) = 84.79, *p* < .001. The HL value was larger than 0.05 (i.e., 0.16), therefore indicating support for the model. Significance level set to *p* < .05

^a^Reference categories—work status: employed

It should be noted that using continuous predictors instead of binary ones did not substantially alter the results presented above. In addition, including all predictors in the multivariate analysis did not substantially alter the results either.

## Discussion

### Predictors of dropout

Our aim was to investigate whether a number of sociodemographic and clinical client factors could predict dropout from a primary care setting, based on indications from previous literature. This had not been studied in the PMHC service context until now. Our results partly support previous findings from the literature that specific sociodemographic factors can predict dropout. These were younger age, being unemployed, lower levels of education, and poor social support. Other sociodemographic factors identified with mixed results in the literature were not significant predictors in this context, such as sex and immigrant background. Contrary to our expectations, clinical factors such as symptom severity, duration of problems, and daily function were not significant predictors of dropout. The overall dropout rate of 25.3% was in accordance with previous rates reported in the literature, notably at the lower end.

#### Age

Our results showed that clients under the age of 30 had a higher risk of dropout, which is in accordance with former research [12, 22, 39, 48, 52]. Fenger et al. [12] explain the link between younger age and dropout by more profound adherence problems and challenges with engagement. Less developed cognitive abilities might reduce the capacity for self-reflection and psychological mindedness, which are beneficial in therapy [1, 34]. Young adulthood is also characterized by less stable social and personal situations [12]. An unpredictable schedule might increase the chance of not showing up. Furthermore, group affiliation becomes more important for self-evaluation. Therefore, feeling different and experiencing stigma can become a barrier to completing therapy. On the contrary, knowledge and access to mental health treatment is more available today compared to previous generations. This might lower the threshold for seeking treatment, while simultaneously lowering the threshold for dropping out when experiencing that treatment does not work. Finally, the described characteristics of younger clients might make it more difficult to establish a good therapeutic alliance, which in itself is a predictor of dropout [22].

#### Level of education

In accordance with previous literature, we found that level of education influenced the likelihood of dropout [12, 52]. Lower levels of education might be linked to dropout on the basis of cognitive abilities, difficulties structuring life, and a low feeling of mastery [7, 12]. Thereby, it might not be education itself that is decisive, but rather the ability to acquire new knowledge. Sharf et al. [45] found in their meta-analysis that the association between therapeutic alliance and dropout was stronger under the condition of lower levels of education. This can be because educated clients are more similar to their therapists, potentially facilitating a good therapeutic alliance [45]. Furthermore, lower levels of education might have secondary consequences such as lower income and poorer working conditions, which can increase perceived life stress. When struggling to meet basic needs, it can be difficult to find time for or remember appointments. Several instances of not showing up in a row, regardless of the cause, might result in treatment rejection, and defining the client as a dropout.

We found in our model that the strength of the relationship between lower levels of education and dropout was somewhat reduced when adding age to the model. The relationship between lower levels of education and dropout might to some extent be explained by age, as more people of younger age are yet to have an education degree.

#### Social support

Poor social support was found to predict dropout, in line with former research [18, 44]. Social support has been identified as an enabling factor for a person's use of healthcare services [1]. Conversely, poor social support can give rise to feeling alone with one's problems and make it more challenging to maintain motivation throughout treatment. These findings underline that the client's ability to show up to treatment is influenced by factors outside the therapist's office.

Another hypothesis is that poor social support can be maintained by the client’s relational patterns. These patterns might be transferred to the therapeutic alliance. Personality traits such as avoidance, hostility, aggressiveness, and low psychological mindedness have been found to negatively influence the therapeutic alliance [1, 22]. A poor therapeutic alliance can subsequently be linked to dropout.

#### Work status

We found that being a student was a statistically significant variable for work status in our first logistic regression analysis. However, when including this variable in the multivariate regression, the significance attenuated. Exploring this further, we found that the relationship between being a student and dropout was reduced when adding age to the model. This is probably due to the fact that students tend to be younger. Based on these results, the possible explanations of the association between dropout and age also applies to the association between being a student and dropout.

We did find an association between dropout and unemployment in this study, which was in line with findings from previous studies [12, 14, 42]. Unemployed clients tend to have lower income levels, which may explain part of the association with drop-out when the therapy is not free of charge. This is not the case for PMHC though and other explanations are therefore warranted. Unemployed clients may on average be less resourceful and may therefore find it more challenging to put in sufficient effort to gain benefit from therapy. It may also be the case that these clients have lower expectations that their own efforts will yield results in therapy. For therapists, it would be worthwhile to be aware of these issues and address them early in therapy.

#### Other findings

We did not find an association between dropout and the remaining sociodemographic factors such as immigrant background and sex. Previous literature has provided somewhat mixed results on these predictors. Furthermore, we did not find any effects for the clinical client variables, contrary to previous research. The lack of association between dropout and high symptom severity might be because our sample is drawn from a primary care service. This entails that the target group was clients with mild to moderate depression and/or anxiety. People with more complex and severe problems were referred to specialized health care. Therefore, the clients in our sample generally had a lower and homogenous symptom severity.

The lack of association between lower symptom severity and dropout might be explained by the nature of the service and the definition of dropout in PMHC. Unlike some other services, PMHC does not follow a given protocol including a set minimum or maximum of sessions for the client. The number of sessions are rather determined by the clients´ needs. Furthermore, the definition of dropout was in our study based on the therapist's evaluation of the treatment goal.

### Study strengths

Our study has several strengths. When collecting the data, questionnaires and measurements were used to cover a wide range of baseline information regarding the clients. With limited missing data (< 3%) and relatively large sample size (*N* = 526), we were able to make thorough analyses with relevant baseline factors identified through the literature.

Our instruments were standardized and validated with acknowledged cut-offs for the central measures of anxiety (GAD-7) and depression (PHQ-9). The only exception was the OSSS-3 with a Cronbach’s alpha of 0.58. This might imply that the instrument lacked some consistency across questions in this sample, and potentially underestimated the association between social support and dropout. The various instruments used in this study are applied within the PMHC service, which makes it possible to compare results from PMHC within and across countries to other similar services such as IAPT. This contributes to a strengthened external validity and generalizability of our results.

When performing the analysis, we included therapists and municipalities as fixed effects. This way, we excluded variations that could be attributed to these factors and thereby reduced the potential for Type I error.

### Study limitations

The results from this study should be considered in the context of some limitations. Firstly, our study only investigates one group of factors, namely client factors. This was due to the nature of our dataset. Client factors alone can not explain dropout, which is rather a complex interplay between the client, therapist, therapeutic alliance, and service [52]. Our results should therefore be supplemented by findings from other groups of factors.

Secondly, our study had limited data on dropout from guided self-help, only used by 1% of our sample. This is a limitation, as guided self-help is an important component of the mixed care model [28]. Thus, this study can not provide solid information about dropout from this treatment modality.

A weakness concerning our understanding of dropout is that we only had the perspective of the therapist at hand. The clients´ experience might have differed from what the therapists reported, thereby weakening the reliability [52].

### Practical implications

Therapists should know that there is an increased risk of dropout among clients of younger age, being unemployed, having lower levels of education or low social support. When therapists identify these predictors, it should encourage them to be more flexible and adaptive to the client. This especially as limited flexibility and individual adjustment from the therapist represents a main reason for dropout [30]. However, therapists often struggle to identify when their interventions are not working, constituting a barrier to being sensitive and flexible [50]. Further on, dropout rates vary substantially between therapists [3, 42].

A way to prevent dropout due to such variations is to seek the client's feedback through formal outcome monitoring systems [17, 50, 54], such as Feedback Informed Treatment (FIT). FIT consists of rating scales monitoring both the client’s improvement and the therapeutic alliance. The tool has been shown to be cost-effective in the context of IAPT [9]. However, FIT only achieves its purpose if it is used correctly [20]. Therefore, implementation of FIT in PMHC must include thorough training on how to utilize results to adapt to the client. To maintain such an implementation over time, it is crucial to establish a feedback culture which should be a leader and service responsibility.

Providing a time perspective of therapy has been found to reduce the risk of dropout [2, 34, 36, 54]. However, giving an absolute time perspective is difficult as sessions in PMHC are based on continuous evaluations of the clients' needs. Therapists can, however, provide an estimate of how many sessions the client can expect, or agree on an “evaluation session” after three appointments.

Forgetting is often mentioned as a reason for not showing up [3, 17]. In our sample 36.1% of the dropout group were terminated because they were unreachable. Pennington and Hudson [37] found lower dropout rates among clients invited by telephone and with a text message reminder, compared to clients invited only by letter. Adapting communication channels to remind clients might engage young people at risk of dropout. However, service routines should also address those who are about to drop out or recently dropped out. Routines for when no-show will lead to discharge are often vague and practices vary between therapists and services [3].

A recent process evaluation of PMHC stresses that the focus on clients’ socioeconomic challenges often has been neglected [28]. It could be argued that socioeconomic challenges should be emphasized more in therapy as a measure to prevent dropout. This aligns well with PMHC’s secondary goal of enhancing work participation. Some clients might even be more in need of work training and social interventions than psychological interventions and should be guided to another service [12].

It is important to remember that dropout is not exclusively negative [29]. Some people leave treatment because they experience improvement already in the first couple of sessions [4, 17]. Others might have low symptom severity to begin with and so they are more ambivalent about treatment [54]. Young people might be overrepresented in this group, as they have a lower threshold for talking about mental health and approaching therapy. Dropout due to early improvement might be especially relevant for primary care services, which aim to be easily accessible and reach people at an early stage. A natural side effect of this strategy is that dropout also becomes an accessible option. Dropout due to early improvement does, however, not guarantee a long-time improvement [4, 34, 54]. Therefore, we need to differentiate problematic cases of dropout from non-problematic cases. It is not realistic to expect dropout-free services. We should rather discuss what kind of dropout is tolerable.

## Conclusions

In conclusion, the present study provides empirical support that is partly in line with previous research on client factors that play a role in predicting dropout from other service settings. The main findings were that people of younger age, unemployed, having lower levels of education, and poor social support had a higher odds ratio of dropping out compared to their reference groups. This had not been studied in the context of PMHC before. Our study provides valuable insight into a large client group who may not get satisfactory effects of treatment. As PMHC has become a national area of investment, this knowledge is of great importance for how we can improve the service to reduce dropout. This can subsequently save both human and economic resources. For future research, it would be beneficial to work towards a unifying definition of dropout, investigate the role of individual therapists and services respectively on dropout, and finally, explore dropout from the clients’ perspective.

## Data Availability

The datasets analyzed during the current study are not publicly available due to ethical restrictions and personal data protection but are available from the corresponding author on reasonable request.

## Abbreviations

CBT: Cognitive Behavioral Therapy
CI: Confidence interval
FIT: Feedback Informed Treatment
GAD-7: Generalized Anxiety Disorder scale
GP: General practitioner
IAPT: Improving Access to Psychological Therapies
NIPH: The Norwegian Institute of Public Health
OR: Odds ratio
NSD: Norwegian Social Science Data Services
OSSS-3: Oslo Social Support Scale
PHQ-9: The Patient Health Questionnaire
PMHC: Prompt Mental Health Care
REK: The regional ethics committee for western Norway
RCT: Randomized Controlled Trial
SES: Socioeconomic status
TAU: Treatment as usual
WSAS: The Work and Social Adjustment Scale
